# Insight into microtubule disassembly by kinesin-13s from the structure of Kif2C bound to tubulin

**DOI:** 10.1038/s41467-017-00091-9

**Published:** 2017-07-10

**Authors:** Weiyi Wang, Soraya Cantos-Fernandes, Yuncong Lv, Hureshitanmu Kuerban, Shoeb Ahmad, Chunguang Wang, Benoît Gigant

**Affiliations:** 1 Department of Central Laboratory, Shanghai Tenth People’s Hospital of Tongji University, School of Life Sciences and Technology, Tongji University, Shanghai, 200092 China; 2 Institute for Integrative Biology of the Cell (I2BC), CEA, CNRS, Univ. Paris-Sud, Université Paris-Saclay, 91198 Gif-sur-Yvette cedex, France

## Abstract

Kinesin-13s are critical microtubule regulators which induce microtubule disassembly in an ATP dependent manner. To clarify their mechanism, we report here the crystal structure of a functional construct of the kinesin-13 Kif2C/MCAK in an ATP-like state and bound to the αβ-tubulin heterodimer, a complex mimicking the species that dissociates from microtubule ends during catalytic disassembly. Our results picture how Kif2C stabilizes a curved tubulin conformation. The Kif2C α4-L12-α5 region undergoes a remarkable 25° rotation upon tubulin binding to target the αβ-tubulin hinge. This movement leads the β5a–β5b motif to interact with the distal end of β-tubulin, whereas the neck and the KVD motif, two specific elements of kinesin-13s, target the α-tubulin distal end. Taken together with the study of Kif2C mutants, our data suggest that stabilization of a curved tubulin is an important contribution to the Kif2C mechanism.

## Abstract (web summary)

Kinesin-13s are microtubule depolymerizing enzymes. Here the authors present the crystal structure of a DARPin fused construct comprising the short neck region and motor domain of kinesin-13 in complex with an αβ-tubulin heterodimer, which shows that kinesin-13 functions by stabilizing a curved tubulin conformation.

## Introduction

Kinesins are microtubule-associated motor proteins that produce a mechanical work associated with ATP hydrolysis. Most kinesins move along microtubules to translocate molecules or organelles^1^ or to slide microtubules apart^2^. In addition, kinesins regulate microtubule dynamics^3^, in particular acting as depolymerases^4^. Kinesins of class 13 (kinesin-13s) are unique depolymerizing enzymes in that they are not motile but target microtubule ends, either directly^5^ or by diffusing along microtubules^6^, to induce disassembly^7^. Unique also is the observation that kinesin-13s are active as monomers^8^, the minimal functional construct comprising the motor domain and the proximal part of a neck region, N-terminal to the motor domain^5^. Moreover, it has been shown that kinesin-13s have an unusual nucleotide cycle that is adapted to their function^5, 9^. Whereas motile kinesins not bound to microtubules are trapped in an ADP-bound state, spontaneous nucleotide exchange occurs at high rate in kinesin-13s allowing them to bind ATP in solution and to start interacting with microtubules in this nucleotide state. Furthermore, although still debated^4^, the current view is that ATP hydrolysis is not required for the depolymerization step per se (removal of tubulin from microtubule ends) but for the recycling step (dissociation of the kinesin from this detached tubulin)^9, 10^.

Sequence and structural analyses have identified several kinesin-13 features likely involved in their specific function. First, kinesin-13s share an elongated L2 loop folded as a β-hairpin, with a conserved lysine-valine-aspartate (KVD) motif at its tip^11, 12^. Mutation studies, targeting the KVD motif residues directly^11, 12^ or indirectly through the shortening of both strands of the L2 hairpin^10^, have demonstrated the necessity of this motif and of its precise positioning for efficient microtubule depolymerization. Structural models, either constrained by electron microscopy or based on the structure of tubulin–motile kinesin complexes, converge in placing the KVD motif at the inter-tubulin longitudinal interface of a protofilament^10, 12, 13^. But these models do differ when it comes to identifying the KVD binding site on tubulin, which therefore remains uncertain.

A second feature of kinesin-13s needed for the depolymerization activity is the neck region, although the more divergent kinesin-13 pKinI from *Plasmodium falciparum* is active as a motor domain construct only^14^. A model wherein the positively charged neck enhances the association rate with microtubules by interacting dynamically with the flexible, negatively charged, C-terminal tails of tubulin has been proposed^15^. In line with this model, the depolymerization efficiency of a kinesin-13 construct made inactive by deleting the distal part of the neck is restored by introducing a stretch of basic residues at the N-terminal end of the protein^16^. These studies highlight the importance of the basic nature of the neck and suggest that a precise conformation of this neck is not required for the function. In partial agreement with this proposal, in the available crystal structures of isolated kinesin-13s, the distal part of the neck is disordered whereas its proximal moiety folds as an α-helix and has been reported to point in two different directions^12, 17^. In contrast, an electron microscopy analysis of dolastatin-induced rings of tubulin decorated by a neck+motor kinesin-13 construct indicates that only half of the motor binding sites are occupied^18^. This observation suggests that, once a kinesin-13 construct is bound, its neck obstructs the neighboring site, hence implying a rather rigid conformation of the neck relative to the motor domain, at least when it is bound to tubulin. Taken together, the structural function of the neck in microtubule depolymerization needs to be defined further.

A third peculiar structural feature of kinesin-13s is the tubulin-binding α4 helix, one of the main kinesin elements that interact with microtubules. In the structure of isolated kinesins, the N-terminal part of this helix is most often disordered and becomes ordered upon microtubule binding. However, in the absence of microtubules, the length of this helix varies, e.g., a longer α4 helix has been observed in kinesin-3 Kif1A^19^. In the case of kinesin-13s, the α4 length is also variable but the same orientation, which is tilted by about 5° compared to ADP-bound Kif1A and 30° compared to ATP-like Kif1A^12^, is found in all the kinesin-13 structures determined so far. This orientation has been proposed to account for the preferential binding of these kinesins to curved tubulin^12^. However, modeling studies have suggested that, upon tubulin binding, the kinesin-13 α4 helix adopts an orientation similar to that found in the tubulin–kinesin-1 complex^10^. Therefore, one needs to determine a higher resolution and experimentally defined structure to specify the binding mode of kinesin-13s to tubulin.

**Fig. 1:**
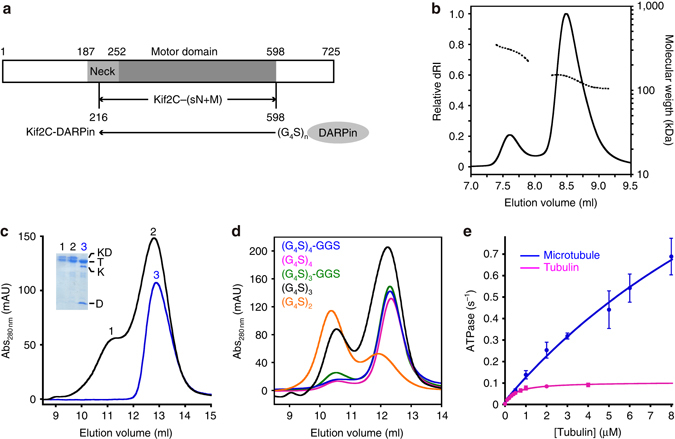
Characterization of Kif2C-DARPin constructs. **a** Schematic presentation of Kif2C, of Kif2C-(sN+M), and of Kif2C-DARPin chimerical constructs. **b** SEC-MALLS analysis of tubulin in complex with Kif2C-(sN+M) linked to the D1 DARPin by a (G_4_S)_3_ peptide. The differential refractive index (normalized dRI, *left axis*) and molecular mass (*right axis*) are plotted as a function of the column elution volume. The derived masses from the static light-scattering data are about 307 kDa for the first peak (elution volume between 7.43 and 7.75 ml) and 153 kDa for the second peak (elution volume 8.2–8.4 ml). These masses reasonably match those calculated from tubulin:construct complexes of stoichiometry 2:2 (about 320 kDa) and 1:1 (about 160 kDa). The profile of the second peak and the mass estimate of its tail (about 110 kDa, elution volume 8.9 to 9.09 ml) are consistent with some dissociation of the complex in the column. Values of the molar mass moments of the peaks are given in Supplementary Table 2. **c** Gel filtration chromatograms of tubulin mixed with a covalent Kif2C-(sN+M)-(G_4_S)_3_-DARPin construct (*black curve*) or with isolated Kif2C-(sN+M) and DARPin proteins (*blue*). For this experiment, 75 mM KCl was added to the elution buffer to reduce Kif2C-induced tubulin aggregation. Inset: analysis of the protein content of the peaks (KD, Kif2C-DARPin construct; T, tubulin; K, Kif2C-(sN+M); D, DARPin). See Supplementary Fig. 9 for an uncropped version of the gel. **d** Gel filtration chromatograms of tubulin in complex with Kif2C-(sN+M) connected to the A-C2 DARPin by the indicated linkers. The construct built with a (G_4_S)_4_-GGS linker gave mainly a 1:1 tubulin:construct complex and led to tubulin–Kif2C-DARPin crystals. **e** Microtubule- and tubulin-stimulated ATPase activity of Kif2C linked to the A-C2 DARPin by a (G_4_S)_4_-GGS peptide. The experimental data points were fitted with the Michaelis–Menten equation. Error bars are s.d. from at least three experiments

We report here the crystal structure of a monomeric functional construct of the human kinesin-13 Kif2C, also named MCAK, in complex with tubulin. The Kif2C construct comprised the motor domain and the C-terminal part of the neck region and has been called “short neck+motor” (Kif2C-(sN+M))^5^ (Fig. 1a). For crystallization purpose, Kif2C-(sN+M) was fused to a designed ankyrin repeat protein (DARPin)^20^ that binds tubulin and prevents its self-assembly^21, 22^. The structural data clarify several features of Kif2C that had remained ambiguous. Altogether with the study of the effect of structure-based designed mutations, these data ultimately provide insights into the structural mechanism of microtubule catalytic disassembly by kinesin-13s.

## Results

### The Kif2C–tubulin structure

In our previous structural studies of (motile) kinesin-1 bound to tubulin^23, 24^, for crystallization purposes, tubulin was stabilized by DARPins that bind its β subunit longitudinal interface^21^. We initially applied the same strategy in the case of kinesin-13s but it failed for a long time, at least in part because of the propensity of these kinesins to aggregate tubulin^5^. To stabilize Kif2C–tubulin–DARPin complexes further, we gathered in the same protein Kif2C-(sN+M) and a DARPin, by linking the C-terminal end of the former to the N-terminal end of the latter, using linkers based on G_4_S repeats (Fig. 1a).

First, we generated a fusion protein between Kif2C-(sN+M) and the D1 DARPin^21^ using a (G_4_S)_3_ linker (Supplementary Table 1). Mixing tubulin with this chimerical construct gave two main species on gel filtration, which are in equilibrium (Supplementary Fig. 1). Size exclusion chromatography coupled to multi-angle laser light scattering (SEC-MALLS) analysis indicated that the mass of the earliest eluting species corresponds to that of a 2:2 tubulin:Kif2C–DARPin complex, whereas the second peak is consistent with a 1:1 complex (Fig. 1b). Given that a tubulin:Kif2C-(sN+M):DARPin mixture (in which the kinesin and the DARPin are not fused) eluted at a position similar to that of the second peak and that the first peak was absent from this chromatogram (Fig. 1c), we concluded that the first peak is a consequence, most likely an artifact, of the Kif2C–DARPin chimeras with inappropriate linker length. Indeed, the relative amount of these two peaks varied according to the length of the linker (Fig. 1d). For these additional experiments, we took advantage of affinity-improved DARPins, which dissociate from tubulin two orders of magnitude slower than the parental one to reach a dissociation constant at equilibrium (*K*_D_) in the subnanomolar range^22^. A construct of Kif2C-(sN+M) fused to the A-C2 DARPin via an optimized linker length of four G_4_S repeats plus three additional amino acids (two Gly and one Ser) gave mainly a 1:1 complex with tubulin (Fig. 1d). This construct, hereafter called Kif2C–DARPin, was chosen for further structural studies.

The basal ATPase activity of Kif2C–DARPin (typically 0.004 ± 0.002 s^−1^) is similar to that of Kif2C-(sN+M) (0.008 s^−1^ (ref. 5)). In addition, its tubulin-stimulated ATPase is close to that of Kif2C-(sN+M) (*k*_cat_ 0.1 ± 0.01 s^−1^ (Fig. 1e) compared to 0.15 s^−1^ (ref. 5)), and the apparent Michaelis–Menten constant remains in the sub-micromolar range (0.5 ± 0.06 μM) even though it increases slightly. These results suggest that the DARPin moiety does not interfere with the folding of Kif2C and that the interactions with tubulin of isolated Kif2C-(sN+M) and of Kif2C in the fusion protein are mostly similar. In contrast, the microtubule-stimulated ATPase indicates a substantial increase of the apparent *K*_m_ (*K*_m_MT) of at least about 15-fold, from 1.13 μM in the case of Kif2C-(sN+M)^5^ to 20 ± 10 μM in the case of Kif2C–DARPin (Fig. 1e), compatible with the DARPin partner interfering with the binding of the Kif2C moiety to a microtubule. There is a slight decrease of the microtubule-stimulated ATPase *k*_cat_ compared to that of Kif2C-(sN+M) but the high *K*_m_MT precluded an accurate *k*_cat_ from being measured.

Crystals of tubulin–Kif2C–DARPin were obtained. The diffraction was anisotropic (about 3.2 Å in two directions, about 4.8 Å in the third direction (Table 1)). Data were therefore processed taking anisotropy into account and the structure was determined by molecular replacement, using tubulin–DARPin and Kif2C as search models. Views of the electron density maps are provided in Supplementary Fig. 2.

**Table 1:** Data collection and refinement statistics

	Kif2C–tubulin–DARPin
*Data collection* ^a^	
Space group	C222_1_
Cell dimensions	
*a*, *b*, *c* (Å)	51.8, 229.8, 293.9
α, β, γ (°)	90.0, 90.0, 90.0
Resolution (Å)	49.0–3.19 (3.38–3.19)
*R*_meas_	0.21 (2.93)
*I*/σ*I*	5.34 (0.43)
CC_1/2_	0.995 (0.342)
Multiplicity	3.56 (3.06)
Completeness (%)	97.3 (85.9)
Anisotropy direction^b^	Resolution where CC_1/2_ > 0.30
overall (Å)	3.26
along *h* axis (Å)	4.83
along *k* axis (Å)	3.21
along *I* axis (Å)	3.19
Completeness after anisotropy correction (%)	66.3 (23.4)
*Refinement*	
Resolution (Å)	42.91–3.19
No. reflections	19903
*R*_work_/*R*_free_	0.211/0.257
No. atoms	
Protein	10299
Ligand	122
Water	0
*B* factors	
Protein	104.2
Ligand	94.6
Coordinate error (Å)	0.48
R.m.s.d.	
Bond lengths (Å)	0.010
Bond angles (°)	1.10
Ramachandran	
Favored region (%)	96.11
Allowed region (%)	3.05
Outliers (%)	0.84

^a^Data were collected on a single crystal. Values in parentheses are for the highest-resolution shell

^b^The anisotropy statistics were computed with AIMLESS^39^

**Fig. 2:**
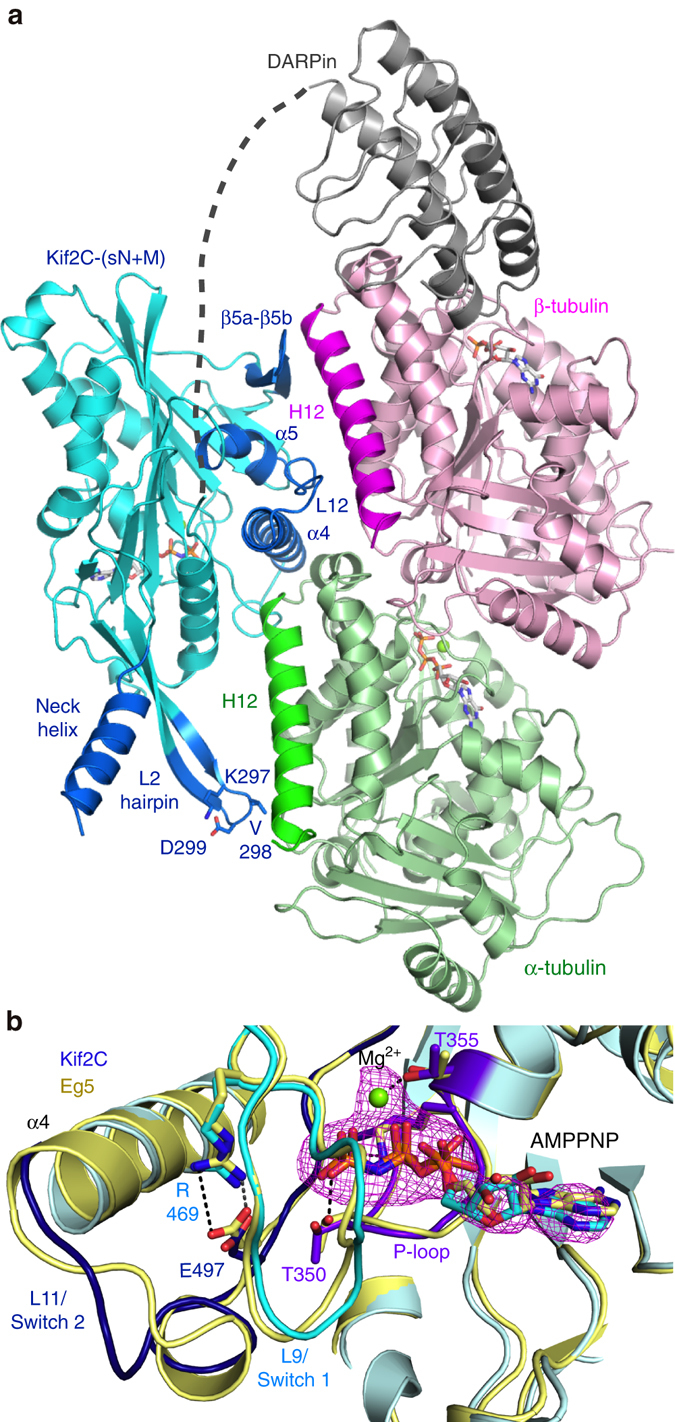
The Kif2C–tubulin complex structure. **a** Overview of the complex crystallized. The (G_4_S)_4_-GGS linker between Kif2C and the DARPin was not defined in the electron density maps and is shown as a *grey dotted line*. See Supplementary Fig. 2 for stereo views of electron density omit maps. **b** Close-up of the nucleotide binding site of Kif2C and comparison with AMPPNP-Eg5 (*yellow*, pdb id 3HQD^26^). The Kif2C nucleotide is presented with its F_obs_–F_calc_ electron density omit map contoured at the 3 σ level. The Kif2C phosphate-binding loops (P-loop, Switch 1 and Switch 2) are rendered each in a different shade of *blue* (*purple blue*, *cyan* and *dark blue*, respectively). Selected interactions are shown as *dotted lines* in the Eg5 structure. See Supplementary Fig. 2f for a stereo version of this figure

The overall conformation of the complex is reminiscent of that of tubulin–kinesin-1^23^. In particular, Kif2C binds at the αβ interface of a curved tubulin heterodimer (Fig. 2a). The tubulin curvature (14.7°) is slightly larger than those observed in the complexes with kinesin-1 (9.2° and 11.6° in the structure with kinesin-1 without nucleotide^24^ and in ATP-like state^23^, respectively), but is similar to values found in other tubulin structures, e.g., in a tubulin–CPAP–DARPin complex^25^ (pdb id 5ITZ). In the complex, Kif2C was bound to the stable ATP analog AMPPNP, leading to a nucleotide binding site conformation at the image of those of the kinesin-5 Eg5 bound to AMPPNP^26^ (Fig. 2b, Supplementary Fig. 2f), of the AMPPNP form of a kinesin-4^27^, and of ATP-like kinesin-1 bound to tubulin^23^. Of note, the L9 loop, which comprises the Switch 1 motif, folded as an extended structure in these different kinesins, suggesting that this conformation is a feature shared in the kinesin superfamily and used for the stabilization of the γ-phosphate nucleotide by conserved Switch 1 residues. In contrast, and different from what is observed in motile kinesins, the L9 loop is poorly ordered in isolated kinesin-13s^12, 17^, which may contribute to the fast spontaneous nucleotide release rate observed in these kinesins^5, 9^. The structure allowed us to identify the Kif2C structural rearrangements upon tubulin binding, the binding site of the Kif2C KVD motif, and the orientation of the neck helix that is required for the Kif2C function. These features will be presented in the next sections.

### Kif2C conformational changes upon tubulin binding

**Fig. 3:**
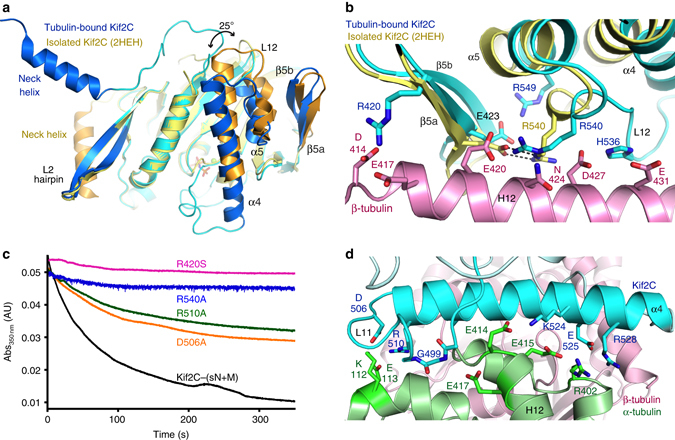
The Kif2C conformational changes upon tubulin binding. **a** Structural changes of the Kif2C tubulin-binding subdomain upon tubulin binding. Kif2C in complex with tubulin is in cyan, with labeled secondary structural elements in *blue* (tubulin is not depicted). Isolated Kif2C (pdb id 2HEH) is in *yellow* with the secondary structural elements that change most upon tubulin binding in *orange*. A superposition taking the P-loop subdomain as a reference is presented as a Supplementary Fig. 3. **b** The restructuring of the β5a–β5b hairpin/α5 helix from isolated ADP-Kif2C (pdb id 2HEH, *in yellow*) to tubulin-bound AMPPNP-Kif2C (*cyan*) places Kif2C Arg420, His536, and Arg540 close to acidic residues of the β-tubulin H12 helix. **c** Mutations of Kif2C residues at the interface with tubulin interfere with the depolymerization activity, as evaluated by a turbidity assay. Taxotere-stabilized microtubules (2 μM) were incubated with 0.2 μM R420S, D506A, R510A, or R540A mutants or with wild-type Kif2C-(sN+M), as indicated. See Supplementary Fig. 5 for additional characterization of the interaction of microtubules with R420S and R540A. **d** The Kif2C–tubulin interface centered on the α4 helix. For clarity, the Kif2C α6 helix was not traced

After superposition of ADP-Kif2C (pdb id 2HEH) on Kif2C in the complex, the overall root mean square deviation (r.m.s.d.) was 1.29 Å (270 Cαs compared). A closer look indicated that the Kif2C elements that deviate most are those that interact with tubulin (Fig. 3a, b), except for the L2 hairpin (see below). These elements correspond mainly to the α4-L12-α5 region and to the short β5a-β5b β-sheet motif and have been gathered in a “tubulin-binding subdomain” in kinesin-1 structural studies^24, 28^. Removing this subdomain from the superposition led to a much better fit (r.m.s.d. 0.59 Å; 218 Cαs compared). As suggested by this low r.m.s.d., the relative orientation of the two other subdomains (named the P-loop and Switch 1/2 subdomains^24^) of Kif2C motor domain did not change much upon tubulin binding (Supplementary Fig. 3).

The large movement of the α4 and α5 helices upon tubulin binding, with a ~25° rotation to adopt a conformation similar to that in tubulin-bound kinesin-1 (Fig. 3a, Supplementary Fig. 4), led also to the reorganization of the α5/β5a–β5b interaction. In particular, Glu423 (in β5a), which interacts with Arg540 (belonging to the L12 loop) in the structure of isolated Kif2C, was displaced towards Arg549 (in α5). This movement freed the kinesin-conserved Arg540 residue and made it available to interact with acidic residues of the β-tubulin H12 helix (Fig. 3b). Consistently, replacing Arg540 by an alanine led to an R540A Kif2C mutant that is unable to depolymerize microtubules (Fig. 3c, Supplementary Fig. 5a). The β5a–β5b movement also placed the N-terminal β5a residue (Arg420) in the vicinity of the N-terminal end of this β-tubulin H12 helix and of the preceding loop (and in particular in the vicinity of Asp414 and Glu417). Arg420 is a relatively well conserved residue in Kif2 proteins (Supplementary Fig. 6), whereas in kinesins of most other classes the nature of the residue at this position is not conserved. To characterize further the contribution of this residue to the Kif2C mechanism, we mutated Arg420 into serine, the residue found at this position in human kinesin-1. We found that the R420S mutant has lost the ability to depolymerize microtubules (Fig. 3c, Supplementary Fig. 5b). This mutation also led to a 10-fold decrease of the microtubule-stimulated ATP hydrolysis rate (Table 2). These results validate β5a–β5b as a zone of interaction with the N-terminal part of the β-tubulin H12 helix, whereas the C-terminal moiety of this helix interacts with the Kif2C L12 loop (Fig. 3b).

**Table 2:** Summary of the properties of the Kif2C-(sN+M) mutants studied

Kif2C protein	Microtubule-stimulated ATPase^a^		Microtubule depolymerase activity^b^
	***k***_cat_ (s^−1^)	***K***_m_MT (μM)	
Kif2C-(sN + M)	3.5 ± 0.1	1.1 ± 0.1	100%
Kif2C-(nsN + M)	2.9 ± 0.1	2.8 ± 0.3	11 ± 4%(6)
Kif2C-(sN + M-Δα)	3.1 ± 0.1	1.0 ± 0.1	17 ± 3% (3)
A233P	n.d.	n.d.	53 ± 5% (2)
R240P	3.6 ± 0.2	1.9 ± 0.4	46 ± 10% (7)
A233P-R240P	2.9 ± 0.2	1.2 ± 0.3	25 ± 7% (2)
M235Q-I236E	2.5 ± 0.2	8.0 ± 1.6	6 ± 2% (3)
L304R	1.6 ± 0.1	2.2 ± 0.4	12 ± 6% (6)
R420S	0.33 ± 0.03	3.3 ± 0.8	5.7 ± 2% (5)
G499A	5.8 ± 0.5	6.0 ± 1.2	90 ± 8% (3)
G499del	1.4 ± 0.1	0.8 ± 0.1	8 ± 8% (4)
A500del	0.97 ± 0.1	0.4 ± 0.1	10 ± 5% (5)
SS-to-G	1.9 ± 0.5	1.1 ± 0.4	26 ± 6% (2)
D506A	1.8 ± 0.2	1.0 ± 0.3	50 ± 10% (4)
R510A	1.5 ± 0.3	17 ± 6	38 ± 12% (5)
R540A	n.d.^c^	n.d.^c^	5.5 ± 3.5% (6)

n.d.: not determined

^a^Kinetic parameters are given as value ± s.e. (calculated from the fit)

**Fig. 4:**
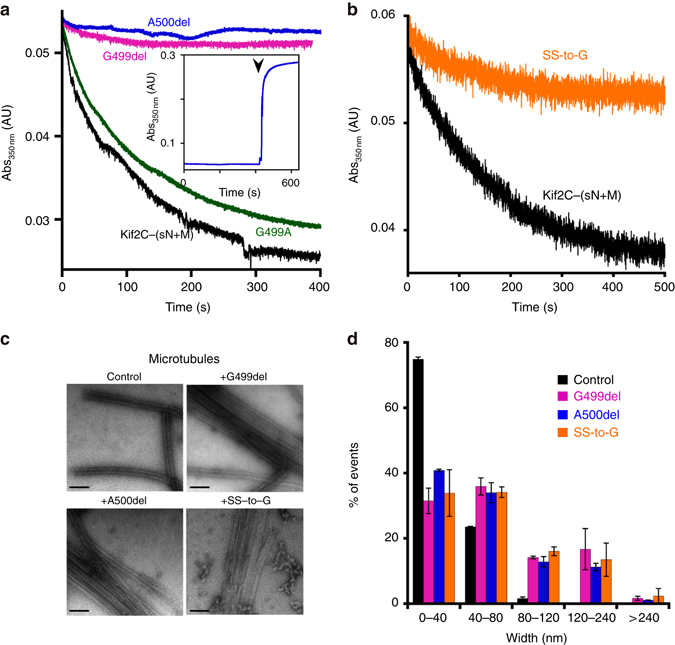
The kinesin-13 specific one-residue insertion in the L11 loop is required for microtubule depolymerization. **a**, **b** Turbidity traces of 2 μM microtubules in the presence of 0.1 μM wild-type Kif2C-(sN+M) or of Kif2C proteins mutated in the L11 loop, as indicated. In the case of deletion mutants, the turbidity signal increased when higher Kif2C concentrations were tested (inset of panel **a**, 1 μM extra A500del added at the time indicated by the *arrow head*). **c** Negative staining electron microscopy images of 2 μM microtubules alone or incubated with 1 μM shortened L11 loop mutants, as indicated. Scale bar, 100 nm. **d** Quantification of the bundling activity. The width of microtubule species (isolated microtubules or bundles) from two independent electron microscopy experiments was measured. Number of measured species: microtubule control, *n* = 90 and *n* = 97 (first and second experiments, respectively); microtubules incubated with G499del, *n* = 87 and *n* = 96; with A500del, *n* = 89 and *n* = 97; with SS-to-G, *n* = 86 and *n* = 95. Error bars are s.d. calculated from these two experiments

**Fig. 5:**
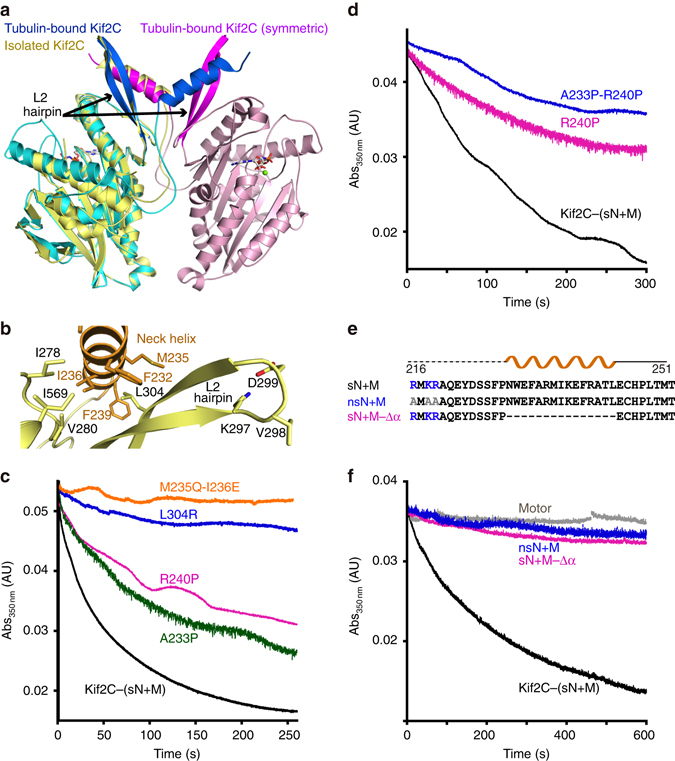
The determinants of the neck that condition Kif2C activity. **a** Neck helix swapping in the tubulin–Kif2C–DARPin crystal. Isolated Kif2C (in *yellow*, pdb id 2HEH) and Kif2C in complex with tubulin (only Kif2C is drawn, in *cyan* with the neck helix and the L2 hairpin highlighted in *blue*) have been superimposed. The motor domain of tubulin-bound Kif2C interacts (*in trans*) with the neck helix of a crystal-related molecule (in *pink*, with the neck helix and L2 in *magenta*). Remarkably, the position of the neck helix of this symmetric Kif2C molecule coincides with that of isolated Kif2C. **b** The environment of Met235, Ile236, and Leu304 shown in the context of the 2HEH structure. **c** Depolymerizing activity of Kif2C constructs with mutated residues at the neck helix–motor domain interface (M235Q-I236E and L304R mutants), or comprising a proline residue in the neck helix. Microtubules (2 μM) were incubated with 0.2 μM wild-type Kif2C-(sN+M) or mutants, as indicated. **d** Turbidity traces of 2 μM microtubules in the presence of 0.1 μM wild-type Kif2C-(sN+M) or of A233P-R240P, as indicated. **e** Sequences of the short neck region of Kif2C and of mutants. The secondary structure is shown above the sequence, with the disordered part shown as a dashed line. **f** Turbidity traces of 1.5 μM microtubules in the presence of 0.1 μM wild-type Kif2C-(sN+M), of Kif2C–Motor (construct starting at residue 252; Fig. 1a), or of Kif2C-(sN+M) neck region mutants, as indicated

^b^based on the turbidity signal variation during the first 50 s of the kinetics and compared to wild-type Kif2C (mean ± s.d. evaluated from *n* = 2–7 replicates, as indicated). Examples of turbidity traces are provided for all mutants in Figs. 3c, 4a,b, 5c,d,f, and in Supplementary Figs. 5, 7 and 8

^c^for this mutant, because of a very weak enhancement of the ATPase rate by microtubules, accurate values could not be determined

The movement and lengthening of α4 (Fig. 3a) led several conserved kinesin-13 residues to point toward tubulin, bringing in particular Arg510 in the vicinity of Glu113 of the tubulin α subunit, Lys524 in that of α-tubulin Glu414 and Glu415, and Glu525 in the vicinity of Arg402 from the same tubulin subunit (Fig. 3d). Consistently, the mutation in kinesin-13s of the residues equivalent to Kif2C Lys524 and Glu525 leads to proteins inactive for the depolymerase activity and with an impaired microtubule-stimulated ATPase^11, 29^. To explain these results, a decrease of the kinesin affinity for microtubules and the loss of the ability to discriminate between microtubule lattice and ends have been proposed^29, 30^. We also studied the R510A mutant. Whereas a recent study has shown that dimeric Kif2C R510A and R510L mutants depolymerize GMPCPP-microtubules as efficiently as the wild-type protein^29^, in our hands, using Taxotere-stabilized microtubules as a substrate, the R510A substitution led to a slight decrease of the depolymerase activity of Kif2C-(sN+M) (Fig. 3c). The mutation led also to a 15-fold increase of the *K*_m_MT compared to wild-type (Table 2), consistent with a lowering of the affinity for microtubules. The D506A mutation, in the L11 Loop, also lowered the depolymerizing activity (Fig. 3c), but in that case the microtubule-stimulated ATPase remained equivalent to that of the parental protein (Table 2). Therefore, the contribution of Asp506, which is close to Lys112 of α-tubulin (Fig. 3d), is likely to be different from that of charged residues of the α4 helix and might be related to that of another specificity of kinesin-13s, a one residue insertion in L11, which is described below.

### An insertion in the L11 loop is important for activity

Upon tubulin binding, as in the case of motile kinesins, the Kif2C L11 loop became fully ordered (Figs. 2b, 3d). Remarkably, in kinesin-13s, the L11 loop is one residue longer than, e.g., in kinesin-1, downstream to the Switch 2 motif^11, 12^. To evaluate the contribution of this insertion to the Kif2C mechanism, we deleted either the Kif2 specific residue Gly499 (G499del mutant) (Supplementary Fig. 6) or the neighboring Ala500 (A500del), or replaced the Ser503-Ser504 motif by one glycine (SS-to-G). We found that the G499del and A500del Kif2C-(sN+M) deletion mutants have lost the ability to depolymerize microtubules and that this activity is substantially reduced in the case of the SS-to-G mutant (Fig. 4a, b, Table 2). These results show that the length of the loop is what matters and not so much the nature of the additional residue. As a reference, a G499A mutant behaved as wild-type Kif2C-(sN+M). Moreover, when the shortened L11 loop mutants in stoichiometric amounts compared to tubulin were tested in the turbidity assay used to monitor microtubule disassembly, instead of a decrease, we observed a signal increase (Fig. 4a, inset) likely resulting from microtubules bundling as indicated by an electron microscopy characterization (Fig. 4c,d).

When these results are considered in light of the structure of the tubulin–Kif2C (sN+M) complex, it is clear that Gly499 may interact with the N-terminal end of the α-tubulin H12 helix (with Glu417 in particular) (Fig. 3d), an interaction that may be prevented by shortening L11. The D506A mutation, which has a milder effect, might proceed similarly by weakening the anchoring point to this tubulin region. Overall, our results suggest that this interaction is important for the catalytic disassembly of microtubules by Kif2C.

### The KVD motif points to the tubulin longitudinal interface

In agreement with previous studies^10, 12^, in the tubulin–Kif2C–DARPin structure, the Kif2C KVD motif at the tip of the L2 hairpin interacts with the distal end of the tubulin α subunit (Fig. 2a), corresponding to the inter-tubulin longitudinal interface of a protofilament. The contribution of the L2 loop leads Kif2C to bury a larger surface with the α subunit (about 1000 Å^2^) than with β-tubulin (about 720 Å^2^), different from kinesin-1 in which the buried surface is more evenly distributed between the two tubulin subunits^23, 24^. At the current resolution, the KVD binding mode is indistinguishable from that inferred from the model we have built based on kinesin-1 bound to tubulin (see Fig. 3 in Ref. ^10^). In particular, the KVD Lys297 residue of Kif2C is in the vicinity of acidic residues (Asp431, Glu434) of the α-tubulin H12 helix. Interestingly, the opposite (N-terminal) end of this helix also interacts with Kif2C, but with L11 and α4 (Fig. 3d). In contrast to other tubulin-interacting elements (Fig. 3a, b), the L2 hairpin did not change orientation upon tubulin binding. Indeed, after superposition of ADP-Kif2C (pdb id 2HEH) on Kif2C in the complex, whereas the overall r.m.s.d. was about 1.3 Å (see above), that of the 16 residues forming the L2 extension (from Val291 to Asn306) was substantially lower (0.66 Å) (Fig. 3a). This observation confirms that the L2 hairpin is an element of the rigid P-loop subdomain in Kif2C (Supplementary Fig. 3), as its shorter version in kinesin-1^24^.

### The neck helix orientation and the depolymerization activity

In the crystal structure, the neck helix pointed toward the solvent and interacted neither with the Kif2C motor domain nor with tubulin (Figs. 2a, 3a). This orientation is different from that observed in the structures of isolated kinesin-13s, in which, most often, the neck helix interacts with the motor domain and in particular with the L2 hairpin (e.g., pdb id 4UBF^17^, 2HEH, 2GRY, see Fig. 3a). However, a crystal contact analysis indicated that similar interactions are also found in the tubulin–Kif2C structure but *in trans*, i.e., between symmetry-related molecules (Fig. 5a). Altogether with a gel filtration analysis that did not show dimerization of monomeric Kif2C-(sN+M), these results suggest that helix swapping was stabilized in the crystal but occurs rarely in solution. Therefore, we propose that the neck helix, although flexible, docks mainly onto the motor domain of the molecule it belongs to.

To validate this hypothesis, we targeted the interaction of the neck helix with the motor domain core and prepared the L304R mutant and the M235Q-I236E double mutant. The Kif2 highly conserved Leu304 residue (Supplementary Fig. 6), which belongs to the L2 hairpin, points to a hydrophobic cavity of the neck helix boxed in by residues Phe232, Met235, Ile236, and Phe239 (Fig. 5b). We reasoned that the L304R mutation should destabilize the L2–neck interaction, as should the mutation of the neck residues Met235 and Ile236, also well conserved and which are at the hydrophobic interface with the motor domain. Whereas these mutants had a microtubule-stimulated ATPase activity that is similar to that of the wild-type protein (Table 2), they were unable to disassemble microtubules (Fig. 5c). Instead, when a higher concentration of L304R mutant was tested in the turbidity assay used to monitor microtubule depolymerization, we observed an increase of the turbidity signal associated with microtubules bundling (Supplementary Fig. 7), as was already the case of the deletion mutants in L11 (Fig. 4). The behavior of L304R suggests that the neck is involved in microtubule bundling. As additional support to this hypothesis, we deleted one residue in the L11 loop of a (neck-less) Kif2C motor domain construct and did not observe any bundling from this construct.

Apart from the L304R and M235Q-I236E mutations, designed to push the neck helix away from the motor domain, we also introduced proline residues in the neck helix to destabilize its helical nature. The corresponding mutants (A233P and R240P) were less efficient microtubule depolymerases, retaining about half of the activity of wild-type Kif2C-(sN + M) (Fig. 5c, Table 2). The activity of a double mutant A233P-R240P was further decreased (Table 2). Interestingly, this activity could only be conveniently measured at low Kif2C protein concentration (e.g., 0.1 μM A233P-R240P; Fig. 5d). Starting from 0.2 μM mutant concentration added to 2 μM microtubular tubulin, a bundling activity took place and this activity predominated at higher A233P-R240P concentrations, as inferred from the variation of the turbidity signal (Supplementary Fig. 8).

Finally, beyond the neck helix, the ca. 12 N-terminal residues of our Kif2C-(sN+M) construct bound to tubulin were not seen in the electron density maps, a feature shared with the structures of isolated kinesin-13s. To precise further the contribution of the disordered part of the short neck, we changed to alanine the three basic residues at the N-terminal end of the protein to produce the “neutralized” Kif2C-(nsN+M) construct (Fig. 5e). This mutant did not depolymerize microtubules (Fig. 5f), consistent with the importance of the basic nature of the neck for the activity^15, 16^. A construct in which the neck helix was removed (sN+M-Δα mutant) was also inactive (Fig. 5e, f). Overall these results highlight the importance of the N-terminal basic residues of the neck and of the positioning of the neck helix for the microtubule depolymerizing activity of Kif2C-(sN + M).

## Discussion

**Fig. 6:**
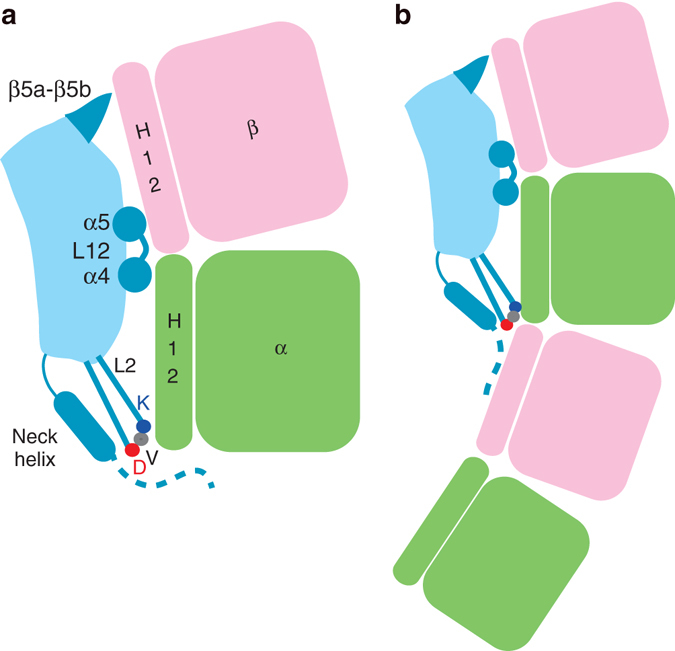
Models of Kif2C stabilizing curved tubulin(s). **a** Kif2C stabilizes curved tubulin by interacting in particular with the N-terminal and C-terminal ends of the H12 helices of both tubulin subunits. The tubulin-interacting elements of Kif2C are in *darker blue*. The KVD motif at the tip of the L2 hairpin is pictured by three dots. The N-terminal part of the short neck region, that is disordered in the structure, is shown as a *dashed line*. **b** Model of Kif2C stabilizing a longitudinal assembly of two tubulins that might be the complex that dissociates from microtubule ends during catalytic disassembly^10^. In this model, the second tubulin molecule is stabilized through interactions with the L2 hairpin tip of Kif2C-(sN+M), and in particular by the aspartate residue (*red dot*) of the KVD motif, and with the N-terminal part of its neck region

We have determined the structure of a functional construct of Kif2C in an ATP-like state and bound to tubulin. The formation of such a complex was shown when microtubule depolymerizing kinesins were initially studied^7^ and is consistent with more extensive biochemical data on another kinesin-13^31^. This complex mimics the species that has been proposed to dissociate from microtubule ends during catalytic disassembly^9^. Our data underscore a major structural rearrangement of the tubulin-binding subdomain of Kif2C, and of the α4-L12-α5 region in particular (Fig. 3). Actually, upon tubulin binding, both the orientations of these helices and the length of α4 became similar to those of kinesin-1 in complex with tubulin (Supplementary Fig. 4) and led to a structural model that is substantially different from previous ones, in which these structural changes were not taken into account^13^. These changes lead kinesin-13 specific α4 residues^11, 29^ to point towards tubulin (Fig. 3d). The structure we determined also confirms the binding site of the KVD motif at the tip of α tubulin, with Lys297 in the vicinity of acidic residues of the H12 helix C-terminal end of this subunit (Fig. 2a). Altogether with the involvement of the other tubulin-interacting elements, including the β5a–β5b hairpin (Fig. 3b), the structural results picture how Kif2C stabilizes a curved tubulin conformation (Fig. 6a). Notably, it interacts with both ends of the H12 helices of the α and of the β tubulin subunits (Fig. 2a). This curved conformation targeted by Kif2C is in the range of those observed in all the crystal structures of unassembled tubulin determined so far, implying that this is a low energy state of non microtubular tubulin^21^. Taken together with the observation that AMPPNP-Kif2C binds more tightly to tubulin than to microtubules^5^, whereas the apparent *K*_m_ of microtubules for the ATPase enhancement of motile kinesins is lower than that of tubulin^23, 32, 33^, our results are in line with a disassembly mechanism driven in part by different affinities for soluble vs. microtubular tubulins.

Our data also clarify the structural role of the neck in the microtubule disassembly mechanism. Whereas the neck helix orientation we found has not been observed previously in structures of isolated kinesin-13s, this orientation likely results from a helix swapping mechanism stabilized in the crystal (Fig. 5a). This hypothesis is supported by mutations aiming to disrupt the interaction of the neck helix with the motor domain, which lead to depolymerization-defective mutants (Fig. 5c). Collectively these data support a model wherein the neck helix interaction with the motor domain is conserved from free Kif2C to Kif2C bound to tubulin (Supplementary Fig. 3). In this last case, the neck helix points toward the distal end of the tubulin α subunit, as does the KVD motif at the tip of the L2 hairpin (Fig. 6a, Supplementary Fig. 4), bringing the neck N-terminal peptide in the vicinity of the longitudinal interface of this tubulin subunit. There, it contributes to the binding affinity of Kif2C for tubulin, because removing the neck region of our Kif2C-(sN+M) construct leads to a 20-fold increase of the corresponding *K*_D_^5^. Because it is disordered in the complex we studied, this N-terminal peptide possibly targets some tubulin flexible parts, e.g., the C-terminal tails^16^, and therefore is expected to remain flexible. Consistently, microtubules treated by subtilisin, which cleaves the tubulin C-terminal tails, are resistant to disassembly induced by the *Xenopus* kinesin-13 XKCM1^34^. Such a dynamic interaction involving the N-terminal peptide is also consistent with the behavior of neck helix mutants. Whereas a helix deletion mutant is inactive, as is also the case of the neck helix-motor domain interface mutants mentioned above, the mutation of neck helix residues to a proline leads to partially active kinesins (Fig. 5, Table 2), suggesting that the neck helix works at least in part as a spacer that brings the N-terminal neck residues close to tubulin. For full activity, though, a precise interaction with the motor domain is required.

We recently proposed that each Kif2C monomer removes two tubulin heterodimers per catalytic cycle of microtubule disassembly^10^. In this framework, the N-terminal part of the neck, whose potential length is sufficient to reach a neighboring tubulin molecule positioned as along a curved protofilament^35^, seems ideally placed to interact with this second tubulin (Fig. 6b). In synergy, the KVD motif would target the longitudinal interface of this assembly, with Asp299 also in position to interact with this neighboring tubulin, in particular with basic residues of the β subunit^10^. These interactions would stabilize a 2:1 tubulin:Kif2C complex which ultimately detaches from microtubule ends. Such a complex is consistent with electron microscopy data showing that kinesin-13s stabilize similar curved protofilament-like assemblies^13, 18^.

Finally, a microtubule bundling activity of Kif2C is unveiled by depolymerization-defective mutants. This activity depends on the presence of the neck, because mutations introduced in a motor-only construct do not lead to bundling. The most likely explanation is that, for bundling, the neck detaches from the motor domain it is bound to and targets a nearby microtubule. Some mutations we have studied were indeed designed to break the interaction between the neck helix and the motor domain (Fig. 5b, c). But other mutations, which inhibit the depolymerization activity and are farther from the neck helix (e.g., mutations in the L11 loop; Fig. 4), also lead to bundling. This observation implies that the interaction of the neck with the motor domain, which is needed for the depolymerization activity, is somehow dynamic. This scheme is reminiscent of what is observed in kinesins from other classes, which comprise a second microtubule binding domain in addition to the motor domain. The two microtubule binding domains may bind to the same microtubule, as proposed here in the case of “functional” Kif2C. Examples include the kinesin-8 Kif18A, where the additional domain is needed to target the microtubule-(+)-end, either to cap this end or to induce disassembly^36^. The two domains may also target different microtubules, leading to crosslinking and bundling as in the case of “impaired” Kif2C. Examples include kinesin-14s, which slide a microtubule with respect to another one^2^.

To sum up, the structure we present enlightens several aspects of the tubulin–kinesin-13 interface and the Kif2C structural changes upon tubulin binding. Remarkably, many of the mutations that annihilate the depolymerizing activity of Kif2C involve residues that contact curved tubulin, as seen in the complex depicted here, suggesting that stabilizing curved tubulin, in particular in the context of the ends of a microtubule, is critical for the depolymerizing activity of Kif2C. More generally, because there are still few high resolution structures of tubulin–kinesin complexes, the tubulin–Kif2C structure will guide the modeling of other such complexes involving depolymerizing but also motile kinesins, some of which also have a depolymerizing activity^3, 4^.

## Methods

### Constructions and protein purification

Kif2C-DARPin chimerical constructs were obtained by connecting by overlap extension PCR the Kif2C-(sN+M) C-terminal end with the N-terminal end of DARPins using different lengths of (G_4_S)_n_-based linker. The primers used as well as the sequence of the DARPin moieties are given in Supplementary Table 1. Mutations in Kif2C-(sN+M) were introduced using the QuickChange site-directed mutagenesis method (Supplementary Table 1). Kif2C-(sN+M) and its mutants were expressed in BL21 Star^TM^ (DE3) *Escherichia coli* cells and purified on a HisTrap FF column followed by a Mono S column (GE Healthcare)^5^. A similar protocol was also used for the Kif2C-DARPin constructs except that a MES-K pH 6.0 buffer was used instead of Pipes-K pH 6.8 for the ion exchange column. Tubulin was purified by two cycles of temperature-dependent microtubule assembly and disassembly^37^.

As a means to check that the Kif2C mutants were properly folded, their microtubule-stimulated ATPase activities were recorded and found to be similar to that of the wild-type protein in most cases (Table 2). The R540A mutant behaved differently, with an ATPase activity that was hardly enhanced in presence of microtubules. To rule out a folding issue for this mutant, we recorded its tubulin-stimulated ATPase (in the presence of DARPin to prevent tubulin oligomerization^5, 10^) and found that it was about 60% of that of Kif2C-DARPin. Altogether with its spontaneous ATP hydrolysis rate, which is similar to that of the wild-type protein, and to its microtubule bundling activity (as inferred from the increase of the turbidity signal; Supplementary Fig. 5a), these results suggest that R540A is folded correctly.

### Size exclusion chromatography

Samples were analyzed on a Superdex 200 10/300 GL column (GE Healthcare) equilibrated with 20 mM Pipes-K, pH 6.8, 1 mM MgCl_2_, 0.5 mM EGTA, and 10 μM AMPPNP. In the case of the gel filtration experiments of Fig. 1c, the elution buffer was supplemented with 75 mM KCl to mitigate Kif2C-induced aggregation of tubulin:Kif2C-(sN+M):DARPin samples.

### SEC-MALLS analysis

SEC was carried out on a Prominence HPLC system (Shimadzu) using a KW803 (Shodex) column run in 20 mM Pipes pH 6.8 buffer containing also 10 μM AMPPNP. Samples of 30 μl at about 3 mg ml^−1^ tubulin–Kif2C–DARPin complexes were run at a 0.5 ml min^−1^ flow rate. Detection was performed using a three-detector static light-scattering apparatus (MiniDAWN TREOS, Wyatt Technology, equipped with a quasi-elastic light-scattering module) and a refractometer (Optilab rEX, Wyatt Technology). Calculations of the molecular weight and estimations of the molar mass moments (Supplementary Table 2) were performed with the ASTRA 6 software (Wyatt Technology) using a dn dc^−1^ value of 0.183 ml g^−1^.

### Crystallization and structure determination

Tubulin–Kif2C–DARPin complexes tested in crystallization were obtained by incubating tubulin-colchicine and AMPPNP-Kif2C-DARPin on ice for 30 min before ultracentrifugation and a purification step on Superdex 200 10/300 gel filtration column (GE Healthcare) in a buffer consisting of 15 mM Pipes-K pH 6.8, 1 mM MgCl_2_, 0.5 mM EGTA, and 10 μM AMPPNP. The tubulin–Kif2C-(sN+M)-(G_4_S)_4_-GGS-DARPin complex was crystallized at 293 K by vapor diffusion in a crystallization buffer consisting of 40 mM Pipes-K pH 6.8 and 4% PEG3350. Crystals were harvested in 80 mM Pipes-K pH 6.8, 8% PEG3350, 1 mM MgCl_2_, 0.5 mM AMPPNP, 10 μM GDP, and 20% glycerol and then flash-cooled in liquid nitrogen.

Data sets were collected at 100 K at the ID23-1 beam line at the European Synchrotron Radiation Facility (Grenoble, France). They were processed with XDS^38^ and AIMLESS^39^ and corrected for anisotropy^40^, using the Staraniso server (http://staraniso.globalphasing.org) developed by Global Phasing Ltd. The structure was solved by molecular replacement with PHASER^41^ using tubulin–A-C2 (pdb id 5EYP) and Kif2C (pdb id 2HEH) as starting models. The structure was refined with BUSTER^42^ with iterative model building in Coot^43^. Data collection and refinement statistics are summarized in Table 1. The relative orientation of the tubulin subunits (the curvature angle) was calculated by superimposing the secondary structural elements of their nucleotide-binding domain^44^. The sequence conservation of kinesin-13s was calculated using the ConSurf program^45^. Figures of structural models were generated with PyMOL (www.pymol.org).

### ATPase measurement

The ATPase activities of Kif2C-(sN+M) mutants and of Kif2C-DARPin were measured at 25 °C using phosphoenolpyruvate, NADH, pyruvate kinase and lactate dehydrogenase in an enzyme-coupled assay^5^. The buffer used for ATPase measurement was 40 mM Pipes-K, pH 6.8, 75 mM KCl, 2 mM MgCl_2_, 1 mM EGTA, 1 mM ATP, and 1 mM DTT. For microtubule-stimulated ATPase measurement, the buffer was supplemented with 20 μM Taxotere to prevent the depolymerization of microtubules by Kif2C constructs.

### Microtubule depolymerization

The microtubule depolymerization activities of Kif2C mutants were evaluated using a turbidity assay^5^. The absorbance at 350 nm was monitored following addition of different concentrations of Kif2C proteins to 1.5 or 2 μM (tubulin concentration) Taxotere-stabilized microtubules, as indicated, at 25 °C and using a buffer consisting of 40 mM Pipes-K pH 6.8, 75 mM KCl, 1 mM EGTA, 2 mM MgCl_2_, 1 mM DTT, and 1 mM ATP. Microtubule disassembly efficiency (Table 2) was estimated from at least two independent experiments and by comparison with a wild-type Kif2C-(sN + M) control obtained in the same conditions (same batch of microtubules, same concentrations of microtubules and of Kif2C proteins).

### Electron microscopy

Taxotere-stabilized microtubules (2 μM) were incubated with 1 μM Kif2C mutants in the presence of ATP for 15 min, then a 10 μl aliquot of the product was applied to a glow-discharged carbon-coated 400-mesh grid for 30 s, and stained with 0.75% (w/v) uranyl formate for 30 s. The grids were observed using a JEM-1230 electronic microscope (JEOL) operated at 80 kV. Microtubule bundling was quantified from two independent experiments by measuring the width of species (microtubules and bundles) from 4 to 6 randomly selected micrographs recorded at a ×20,000 magnification.

### Data availability

Coordinates and structure factors of the tubulin–Kif2C-DARPin structure have been deposited in the Protein Data Bank with accession code: 5MIO (Table 1). Data supporting the findings of this study are available from the corresponding authors upon reasonable request.

## Electronic supplementary material

Supplementary Information
