# Supplementary material for: Insight into microtubule disassembly by kinesin-13s from the structure of Kif2C bound to tubulin
Source: Nat Commun. 2017 Jul 10;8:70. doi: 10.1038/s41467-017-00091-9 (PMC5503940; doi:10.1038/s41467-017-00091-9)
Supplement: Supplementary file 1 — Supplementary Information [file 41467_2017_91_MOESM1_ESM.pdf]

File name: Supplementary Information
Description: Supplementary figures, supplementary tables and supplementary references.

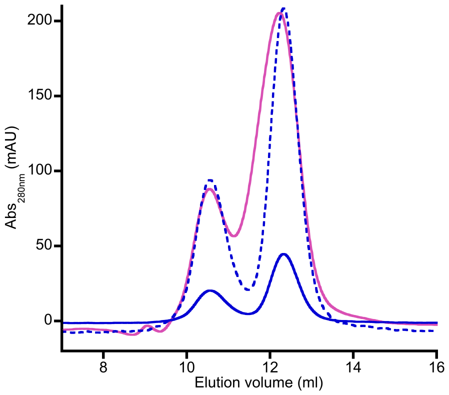

Supplementary Figure 1. The complexes that tubulin makes with Kif2C-DARPin proteins are in equilibrium. A mixture of tubulin and of Kif2C-(sN+M) linked to the D1 DARPin by a $(G_4S)_3$ peptide was analyzed by gel filtration (magenta). The second and main peak of this first injection was collected and analyzed on the same column (blue curve). The absorbance signal of the second run is also shown on a different scale (dotted line) for a better visualization of the relative amount of the two peaks compared with that of the first run. The two peaks of the second run are better defined, possibly because of a smaller amount of loaded protein.

a

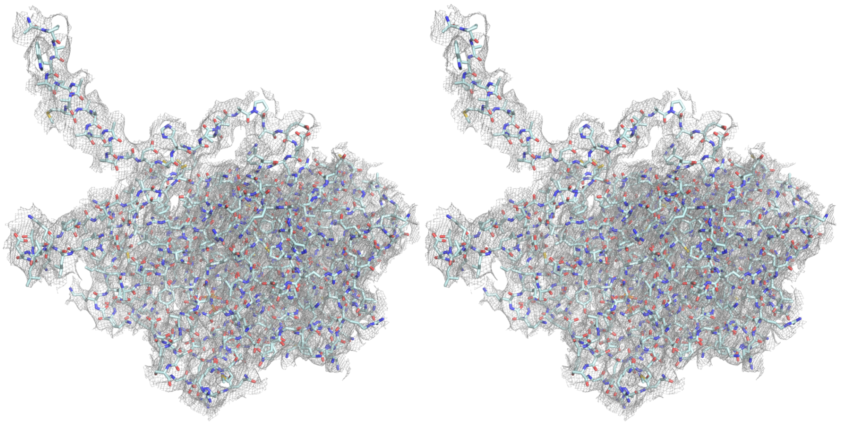

b

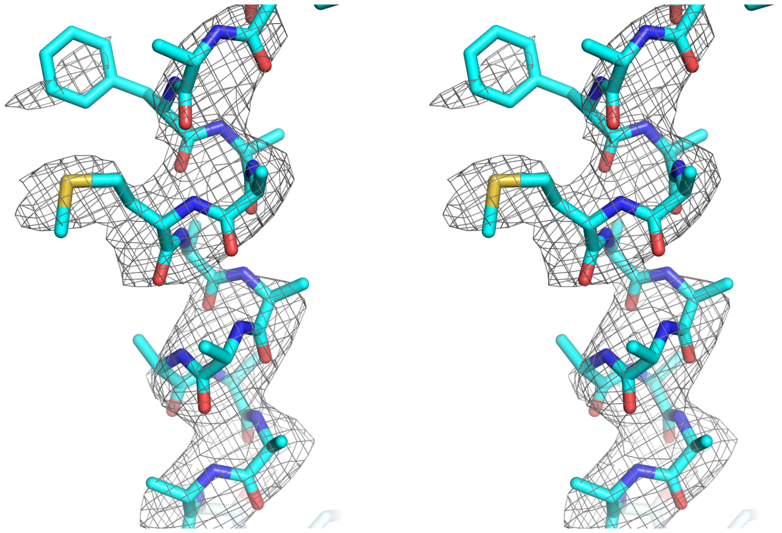

c

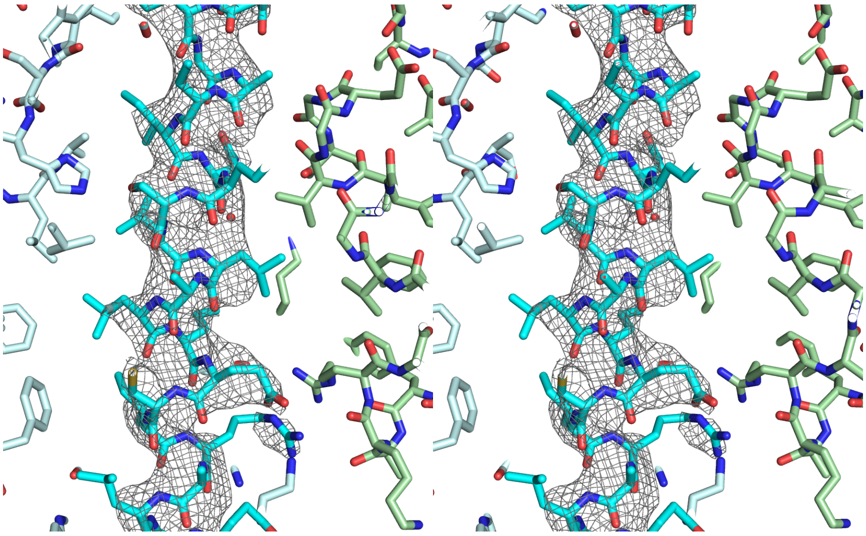

d

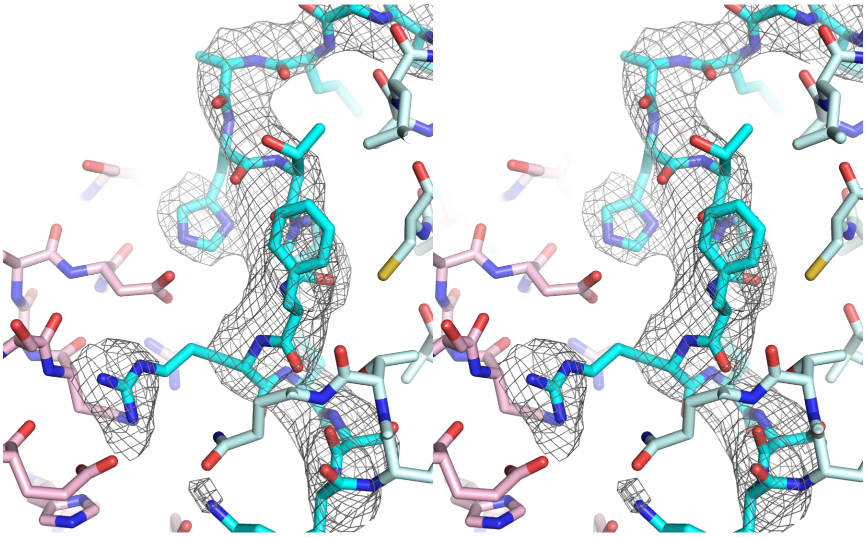

e

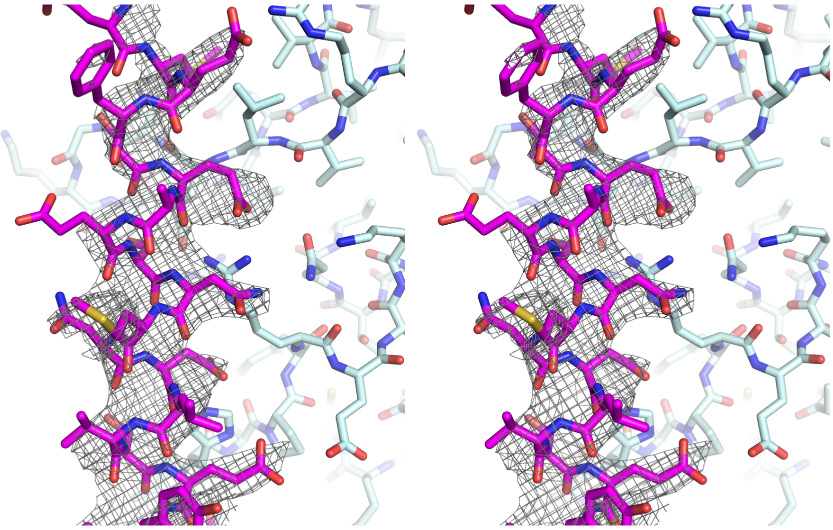

f

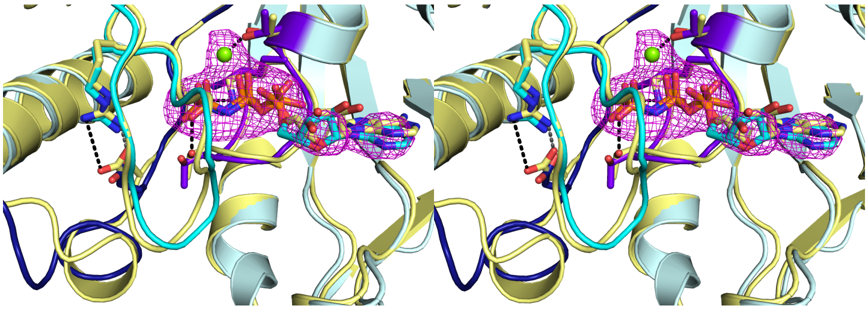

Supplementary Figure 2. Stereo views of tubulin–Kif2C-DARPin omit maps. (**a**) Composite omit map calculated with PHENIX[1] over the whole structure and displayed on Kif2C. It is contoured at the 1 σ level. (**b-e**) F_{obs}-F_{calc} omit maps calculated with BUSTER[2] and contoured at the 3 σ level. Kif2C is in cyan, α-tubulin in green, and β-tubulin is pink. The region that has been removed to calculate the omit map is shown in brighter color. (**b**) Kif2C neck helix. Whereas the electron density for most of the side chains is weak and these side chains have not been traced, some residues (e.g. Met235) are clearly defined. (**c**) Kif2C α4 helix. (**d**) Kif2C L12 loop. (**e**) H12 helix of β-tubulin. In this case, for clarity, only the H12 helix (which has been removed from the model to calculate the omit map) is shown for β-tubulin. (**f**) Stereo version of Fig. 2b.

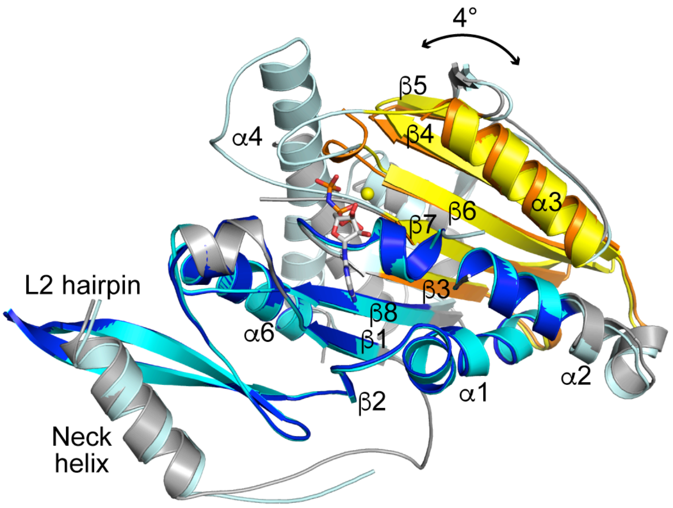

Supplementary Figure 3. Kif2C P-loop and Switch 1/2 subdomains do not reorient much upon tubulin binding. The P-loop subdomains of ADP-Kif2C (pdb id 2HEH) and of tubulin-bound Kif2C have been superimposed (r.m.s.d. after superposition 0.33 Å; 99 Cαs compared). To match the Switch 1/2 subdomains in these two structures, a 4° rotation of one Switch 1/2 block with respect to the other one is needed (Switch 1/2 subdomain r.m.s.d. after superposition: 0.67Å; 68 Cαs compared). The subdomains were defined taking those of human kinesin-1 as a guide[3]. Color code: ADP-Kif2C is in grey with the P-loop subdomain in cyan and the Switch 1/2 block in orange; tubulin-bound AMPPNP-Kif2C is in pale cyan, with the P-loop subdomain in blue and the Switch 1/2 subdomain in yellow. The neck helix depicted for tubulin-bound Kif2C is that of a symmetric molecule, believed to be the active conformation of this neck helix (see text), and coincides with the one observed in the 2HEH structure.

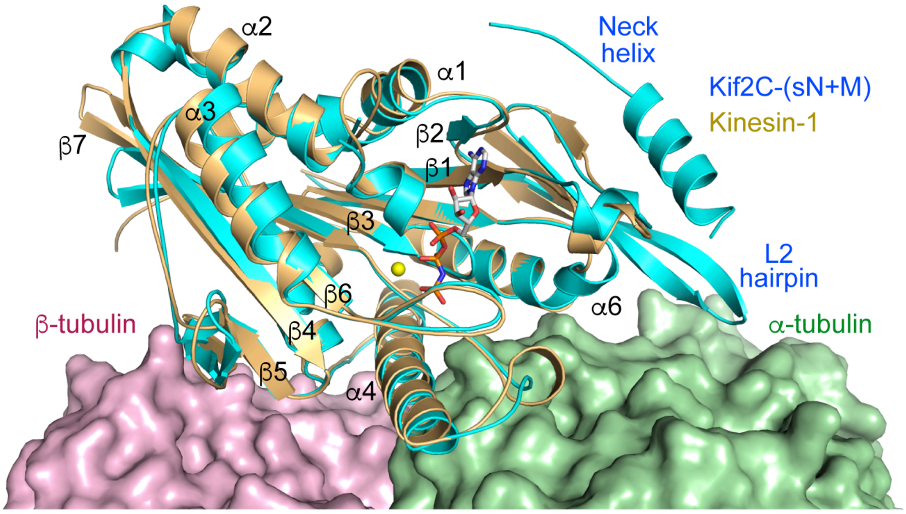

Supplementary Figure 4. Kif2C and kinesin-1 in their ATP-like form adopt a similar conformation upon tubulin binding. AMPPNP-Kif2C (this work) and ATP-like kinesin-1 (pdb id 4HNA[4]) have been superimposed (r.m.s.d. after superposition: 1.23 Å; 302 Cαs compared). Kif2C is in cyan, kinesin-1 in light brown. Only the Kif2C nucleotide is drawn. As in Supplementary Fig. 3, in the case of Kif2C, the neck helix depicted is that of a crystal-symmetry related molecule. Tubulin from tubulin–Kif2C-DARPin is shown.

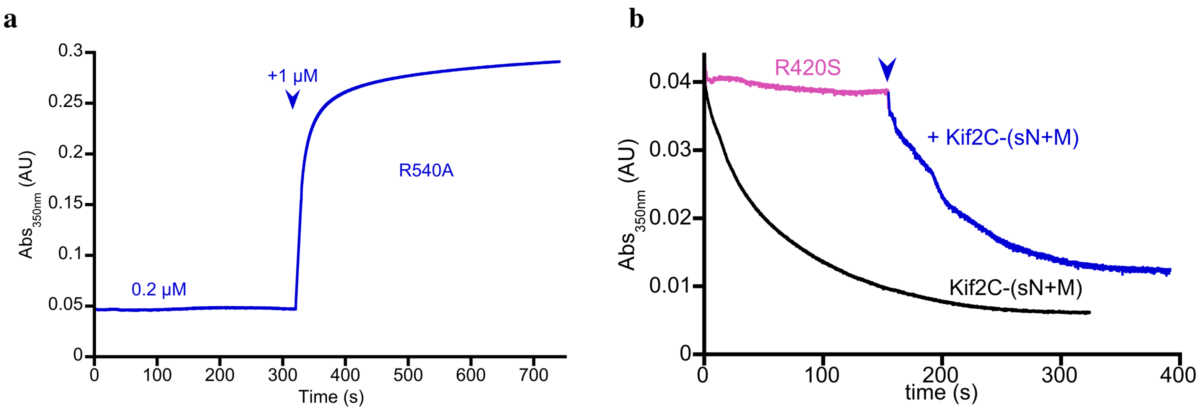

Supplementary Figure 5. Additional characterization of the R540A and R420S Kif2C mutants (related to Fig. 3c). (a) Taxotere-stabilized microtubules (2 µM) were mixed with 0.2 μM R540A. After about 320 s (arrow head), 1 µM extra R540A were added, leading to a turbidity signal increase. **(b)** Turbidity traces of 2 µM microtubules after addition of 0.2 µM wild type Kif2C or its R420S mutant. In this last case, 0.2 µM wild type Kif2C were added after about 150 s (blue arrow head) as an additional positive control for microtubule disassembly.

```
   220       230       240       250       260       270       280       290
* **|         |  * **   *         |         |         |         |         |
RMKRAQEYDSSFPNWEFARMIKEFRATLECHPLTMTDPIEEHRICVCVRKRPLNKQELAKKEIDVISIPSKCLLLVHEPK
             -- neck helix--               ---β1--                      ------L2

   300       310       320       330       340       350       360       370
    |   *     |         |         |         |         |         |         |
LKVDLTKYLENQAFCFDFAFDETASNEVVYRFTARPLVQTIFEGGKATCFAYGQTGSGKTHTMGGDLSGKAQNASKGIYA
 hairpin-------  -β2    ---------α1--------- ---β3--[P-loop]α2a     L5      ---

   380       390       400       410       420       430       440       450
    |         |         |         |         *         |         |         |
MASRDVFLLKNQPCYRKLGLEVYVTFFEIYNGKLFDLLNKKAKLRVLEDGKQQVQVVGLQEHLVNSADDVIKMIDMGSAC
-α2b-------         ----β4----  -β5-        -β5A-   -β5B-    -β5  ------α3------

   460       470       480       490       500       510       520       530
    |         |         |         |        **  ** *   *         |         |
RTSGQTFANSNSSRSHACFQIILRAKGRMHGKFSLVDLAGNERGADTSSADRQTRMEGAEINKSLLALKECIRALGQNKA
-   L9    [Sw 1]---β6---    ---β7---[Sw 2]   L11    ------------α4------------

   540       550       560       570       580       590
    *         |         |         |         |         |
HTPFRESKLTQVLRDSFIGENSRTCMIATISPGISSCEYTLNTLRYADRVKELSPHSGPSGEQ
 L12   ----α5-----   ---β8----    --------α6--------
```

Consurf code:

```
1  2  3  4  5  6  7  8  9
Variable   Average   Conserved
```

X - Insufficient data (the calculation for this site was performed on less than 10% of the sequences).

Supplementary Figure 6. Sequence conservation in kinesin-13s. Sequence conservation was calculated by the Consurf webserver (http://consurf.tau.ac.il/2016/). Scores range from 1 (not conserved, blue) to 9 (highly conserved, magenta) according to the Consurf color code[5]. The analysis is based on 150 sequences retrieved from the UniProt database and having a maximum of 95% sequence identity with human Kif2C-(sN+M) taken as a reference. No sequence from the Kif24 subgroup of kinesin-13s was retrieved. About 60% of the selected sequences were of vertebrate Kif2 proteins (among which about 50% were of mammalian kinesins) and there were about 27% of kinesin-13 sequences from arthropods. The sequence conservation presented here is broadly consistent with those found by others[6,7]. The residues that have been mutated in this study are marked by an asterisk (with the exception of the neck helix deletion mutant). In addition, Arg330 and Arg379 have been mutated to Ala in all the Kif2C constructs to diminish protein aggregation in the presence of tubulin[8,9].

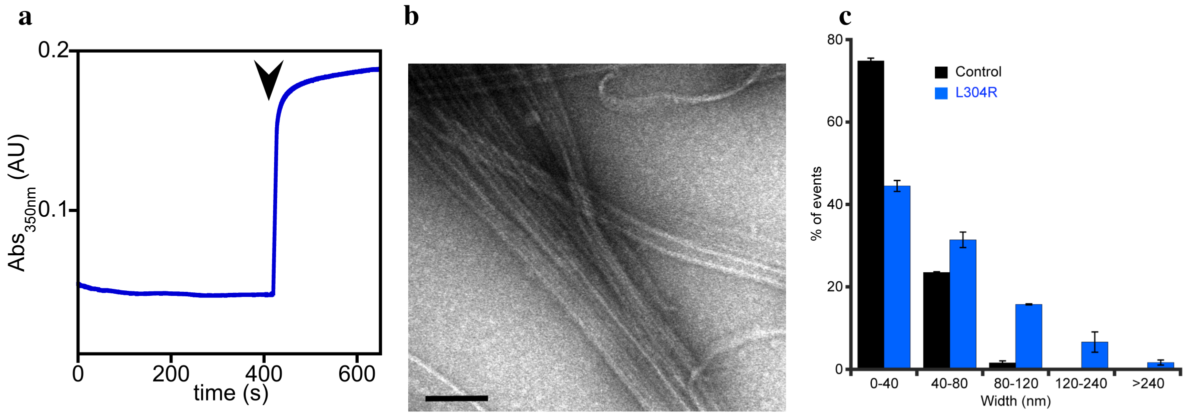

Supplementary Figure 7. The Kif2C L304R mutant induces the bundling of microtubules. (a) Turbidity signal variation of 2 µM microtubules incubated with 0.2 µM L304R. After about 400 s (arrow head), 1 µM extra L304R were added. (**b**) Negative staining electron microscopy images of 2 µM microtubules incubated with 1 µM L304R for 15 min. Scale bar, 100 nm. (**c**) Bundling statistics. The control (Taxotere-stabilized microtubules without Kif2C protein) is taken from Fig. 4d. Error bars are s.d. deduced from two independent experiments (n=89 and n=97).

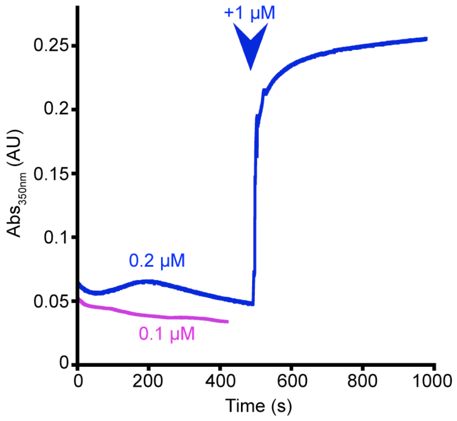

Supplementary Figure 8. The A233P-R240P mutant has both depolymerization and bundling activities. Turbidity traces of 2 µM microtubules are shown. The depolymerization activity is best seen at low mutant concentration (0.1 µM, magenta). At 0.2 µM A233P-R240P, the turbidity signal hardly decreases (blue curve) and this signal increases when 1 µM extra A233P-R240P is added (blue arrow head).

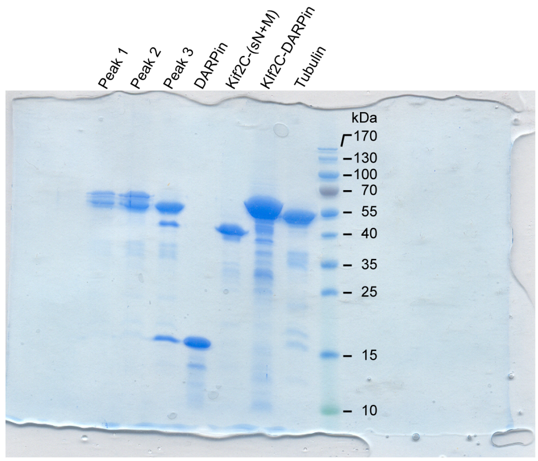

Supplementary Figure 9. Uncropped version of the gel shown in Fig. 1c.

Supplementary Table 1. Primers used for PCR and mutagenesis in this study.

Kif2C-DARPin constructs[a]		
	Forward primer	Reverse primer
Common for all constructs	CTGGCAGCCCATGGGAAGAAT GAAGAGAGCTCAGGAG	TGCTCTGCCTCGAGATTAAGCTTTTGCAGGATTTC
Linker length: $(G_4S)_2$	GGAGGAGGTGGATCTGGTGGTGGAGGTTCT GACCTGGGTAAGAAACTGCTG	AGAACCTCCACCACCAGATCCACCTCCTCC CTGCTCTCCACTGGGCCCACT
$(G_4S)_3$	GGAGGAGGTGGATCTGGTGGTGGAGGTTCTGGA GGTGGAGGTTCTGACCTGGGTAAGAAACTGCTG	AGAACCTCCACCTCCAGAACCTCCACCACCAGA TCCACCTCCTCCCTGCTCTCCACTGGGCCCACT
$(G_4S)_3GGS$	GTTCTGGAGGATCAGACCTGGGTAAGAAACTGCTG	TTACCCAGGTCTGATCCTCCAGAACCTCCA CCTCCAGAACC
$(G_4S)_4$	GTTCTGGAGGTGGTGGATCAG ACCTGGGTAAGAAACTGCTG	AGGTCTGATCCACCACCTCCAGAACCTCCA CCTCCAGAAC
$(G_4S)_4GGS$	GATCTGGCGGAAGTGACCTGGGTAAGAAACTGCTG	TTACCCAGGTCACTTCCGCCAGATCCACCA CCTCCAGAACCAC
Kif2C-(sN+M) mutants		
Kif2C-(nsN+M)	ATGATAGCCCATGGGAGCAATGGCAGCAGCTCAG GAGTATGACAGTAGTTTTCCAAAC	TGCTATGCCTCGAGCTGCTCTCCAC TGGGCCCACT
Kif2C-(sN+M-Δα) (2steps)	GCTCAGGAGTATGACAGTAGTTTTCCAGAATGTCA TCCACTTACTATGACTG	TGCTATGCCTCGAGCTGCTCTCCAC TGGGCCCACT
	ATGATAGGCCATGGGAAGAATGAAGAGAGCTCA GGAGTATGACAGTAGT	
A233P	CCAAACTGGGAATTTCCGCGAATGATTAAAGA ATTTCGGGC	GCCCGAAATTCTTTAATCATTCGCGGAAATTC CCAGTTTGG
R240P	GCCCGAATGATTAAAGAATTTCCGGCTACTTTGGA ATGTCATCC	GGATGACATTCCAAAGTAGCCGGAAATTCTTTAAT CATTCGGGC
A233P-R240P	CCAAACTGGGAATTTCCGCGAATGATTAAAGAA TTTCCG	CGGAAATTCTTTAATCATTCGCGGAAATTCCCA GTTTGG
M235Q-I236E (2steps)	GGGAATTTGCCCGAATGGAGAAAGAATTT CGGGCTAC	GTAGCCCGAAATTCTTTCTCCATTCGGGC AAATTCCC
	CTGGGAATTTGCCCGACAGGAGAAAGAATTTCGG	CCGAAATTCTTTCTCCTGTCGGGCAAATTCCCAG
L304R	GACTTAACAAAGTATCGGGAGAACCAAGCATTC	GAATGCTTGGTTCTCCCGATACTTTGTTAAGTC
R420S	CAAGAAGGCCAAGCTGAGCGTGCTGGAGGACG	CGTCCTCCAGCACGCTCAGCTTGGCCTTCTTG
G499A	GGAATGAGCGAGCCGCGGACACTTC	GAAGTGTCCGCGGCTCGCTCATTCC
G499del	GGAATGAGCGAGCGGACACTTCCAG	CTGGAAGTGTCCGCTCGCTCATTCC
A500del	GAATGAGCGAGGCGACACTTCCAGTGC	GCACTGGAAGTGTCGCCTCGCTCATTC
SS-to-G	CGAGGCGCGGACACTGGCGCTGACCGGCAG	CTGCCGGTCAGCGCCAGTGTCCGCGCCTCG
D506A	CACTTCCAGTGCTGCCCGGCAGACCC	GGGTCTGCCGGGCAGCACTGGAAGTG
R510A	ACCGGCAGACCGCCATGGAGGGCGCAGAAAT	ATTTCTGCGCCCTCCATGGCGGTCTGCCGGT
R540A	GCTCACACCCCGTTCGCTGAGAGCAAG CTGACACAG	CTGTGTCAGCTTGCTCTCAGCGAACGG GGTGTGAGC

[a] The amino acid sequence of the DARPin partners fused to Kif2C-(sN+M) is:

- construct based on D1 DARPin[10]:

```
DLGKKLLEAARAGQDDEVRILMANGADVNATDASGLTPLHLAATYGHLEIVEVLLKHGADVNAIDIMGSTPLHLA
ALIGHLEIVEVLLKHGADVNAVDTWGDTPLHLAAIMGHLEIVEVLLKHGADVNAQDKFGKTAFDISIDNGNEDLA
EILQKLNLEHHHHHH
```

- constructs based on A-C2 DARPin[11]:

```
DLGKKLLEAARAGQDDEVRVLMANGADVNATDASGLTPLHLAATYGHLEIVEVLLKHGADVSASDLMGSTPLHLA
ALIGHLEIVEVLLKHGADVNAVDTWGDTPLRLAAVMGHLKIVEALLKHGADVNAQDKFGKTAYDTSIDNGSEDLA
EILQKLNLEHHHHHH
```

Supplementary Table 2. Molar mass moments of the SEC-MALLS experiment of Fig. 1b. They are given in g/mol ± error (which represents the statistical consistency of the data).

Moments	Mw	Mn	Mp	Mz
First peak				
7.43 – 7.75 ml	3.069×10^5±0.54%	3.062×10^5±0.54%	3.073×10^5±0.44%	3.076×10^5±1.2%
Second peak				
8.2 – 8.4 ml	1.534×10^5±0.3%	1.534×10^5±0.3%	1.526×10^5±0.13%	1.534×10^5±0.67%
8.9 – 9.09 ml	1.084×10^5±1.79%	1.084×10^5±1.79%	1.100×10^5±1.31%	1.084×10^5±3.99%
8.2 – 9.09 ml	1.372×10^5±0.47%	1.353×10^5±0.57%	1.483×10^5±0.21%	1.389×10^5±1.0%

Supplementary References

1 Adams, P. D. *et al*. PHENIX: a comprehensive Python-based system for macromolecular structure solution. *Acta Crystallogr D Biol Crystallogr* **66**, 213-221 (2010).

2 Bricogne, G. *et al*. BUSTER version 2.10.0 Cambridge, United Kingdom: Global Phasing Ltd. (2011).

3 Cao, L. *et al*. The structure of apo-kinesin bound to tubulin links the nucleotide cycle to movement. *Nat Commun* **5**, 5364 (2014).

4 Gigant, B. *et al*. Structure of a kinesin-tubulin complex and implications for kinesin motility. *Nat Struct Mol Biol* **20**, 1001-1007 (2013).

5 Landau, M. *et al*. ConSurf 2005: the projection of evolutionary conservation scores of residues on protein structures. *Nucleic Acids Res* **33**, W299-302 (2005).

6 Shipley, K. *et al*. Structure of a kinesin microtubule depolymerization machine. *EMBO J* **23**, 1422-1432 (2004).

7 Patel, J. T. *et al*. The family-specific α4-helix of the kinesin-13, MCAK, is critical to microtubule end recognition. *Open Biol* **6**, 160223 (2016).

8 Tan, D., Rice, W. J. & Sosa, H. Structure of the kinesin13-microtubule ring complex. *Structure* **16**, 1732-1739 (2008).

9 Wang, W. *et al*. Kif2C minimal functional domain has unusual nucleotide binding properties that are adapted to microtubule depolymerization. *J Biol Chem* **287**, 15143-15153 (2012).

10 Pecqueur, L. *et al*. A designed ankyrin repeat protein selected to bind to tubulin caps the microtubule plus end. *Proc Natl Acad Sci U S A* **109**, 12011-12016 (2012).

11 Ahmad, S. *et al*. Destabilizing an interacting motif strengthens the association of a designed ankyrin repeat protein with tubulin. *Sci Rep* **6**, 28922 (2016).
